# Silicon rich nitride ring resonators for rare – earth doped telecommunications-band amplifiers pumped at the O-band

**DOI:** 10.1038/s41598-017-09732-x

**Published:** 2017-08-22

**Authors:** P. Xing, G. F. R. Chen, X. Zhao, D. K. T. Ng, M. C. Tan, D. T. H. Tan

**Affiliations:** 1 Photonics Devices and Systems Group, Singapore University of Technology and Design, 8 Somapah Rd, Singapore, 487372 Singapore; 2 Engineering Product Development, Singapore University of Technology and Design, 8 Somapah Rd, Singapore, 487372 Singapore; 3 Data Storage Institute (A*STAR) Agency for Science Technology & Research, 2 Fusionopolis Way #08-01 Innovis, Singapore, 138634 Singapore

## Abstract

Ring resonators on silicon rich nitride for potential use as rare-earth doped amplifiers pumped at 1310 nm with amplification at telecommunications-band are designed and characterized. The ring resonators are fabricated on 300 nm and 400 nm silicon rich nitride films and characterized at both 1310 nm and 1550 nm. We demonstrate ring resonators exhibiting similar quality factors exceeding 10,000 simultaneously at 1310 nm and 1550 nm. A Dysprosium-Erbium material system exhibiting photoluminescence at 1510 nm when pumped at 1310 nm is experimentally demonstrated. When used together with Dy-Er co-doped particles, these resonators with similar quality factors at 1310 nm and 1550 nm may be used for O-band pumped amplifiers for the telecommunications-band.

## Introduction

The discovery of rare earth doped fiber amplifiers two decades ago resulted in the proliferation of the 1310 nm and 1550 nm wavelengths in telecommunications^1, 2^. Long – haul data transmission over optical fibers were made possible with periodic amplification using erbium doped fiber amplifiers followed by optical regeneration. In such fiber amplifiers, a pump at a different wavelength than that being amplified is used to amplify light. In erbium doped fiber amplifiers (EDFAs), a pump at 980 nm is commonly used^3, 4^. For silicon photonic integrated circuits (PIC), waveguide amplifiers are part of the photonics toolkit needed to realize sophisticated systems similar to those which already exist in fiber platforms. Ways to achieve light amplification in silicon PIC includes hybrid integrating III-V semiconductor gain media with silicon photonic devices^5–9^ and waveguide amplifiers utilizing rare-earth-ions doped dielectric materials as the waveguide core^10–15^ or cladding^16, 17^. Compared with the electrically pumped III-V semiconductor amplifier, the optically pumped rare earth ions doped waveguide amplifiers usually provide much lower gain per unit length^18^. However, fabrication of III-V semiconductor amplifiers is much more complicated than fabrication of rare-earth-ions doped waveguide amplifiers. More complicated fabrication process can lead to low yield and high cost. Also, III-V semiconductor amplifiers exhibit very bad temperature stability. Thus, the rare-earth-ions doped waveguide amplifiers have some advantages over III-V semiconductor amplifiers in some specific applications. The most commonly used host material for rare-earth-ions doping are glass^10, 11^, LiNbO_3_^10^ and Al_2_O_3_^12–17^. Al_2_O_3_ is getting more and more attention due to its high rare-earth-ions solubility, broad transparency spectrum, and large deposition flexibility. The larger refractive index of Al_2_O_3_ (1.65) compared to silica (1.45) makes it suitable for waveguide core material^12–15^. Also, when integrating with silicon nitride waveguide^16, 17^, it can be used as cladding material to further reduce the fabrication complexity. To date, the most common platform for amplifying light at 1550 nm involves the use of a 980 nm pump. An alternative configuration for light amplification at 1550 nm involves using a pump at 1310 nm. This configuration involves a different material system of Dysprosium-Erbium (Dy-Er) codoped particles to achieve downconversion to 1550 nm.

In many sub-fields of photonics, resonators are used to reduce the threshold power required to observe a specific phenomenon. Resonators are utilized for low power optical parametric amplification^19, 20^, lasing^21, 22^, optical switching^23–25^, sensing of low analyte concentrations^26, 27^ and optical buffers^28^. With regards to optical amplifiers, ring resonators could be used to reduce the pump power required for signal amplification. Such an approach requires that the resonator operates sufficiently well at both the pump and signal wavelengths. However, it is often thought that higher order modes are excited either during light coupling from a fiber, or around waveguide bends akin to those in ring resonators. Therefore, resonators which exhibit good resonant behavior for the fundamental mode with minimal losses from higher order modes are of great merit.

In conventional Er-doped amplifiers amplifying light at 1550 nm, the pump wavelength is at 980 nm. Because the pump wavelength is far from the signal wavelength, any waveguide or resonator designed for the signal at 1550 nm would not be optimized for use at 980 nm. Utilization of a pump closer to the 1550 nm wavelength is preferable for several reasons. First, the number of modes existing within a waveguide designed to be single mode at 1550 nm will be larger at 980 nm compared to 1310 nm. This leads to issues such as modal dispersion and increased propagation losses from higher order modes. Secondly, the 1310 nm wavelength is still within the telecommunications band, and is arguably an easier laser wavelength to access, manipulate and combine with the 1550 nm light. Thirdly, coupling strengths between the bus waveguide and ring would not be easy to optimize for critical coupling because of the different mode profiles at each wavelength. Consequently, waveguide amplifiers pumped at 1310 nm have several advantages over those pumped at 980 nm. An alternative configuration for light amplification at 1550 nm involves using a pump at 1310 nm. This configuration involves a different material system Dy-Er co-doped particles to achieve down conversion to 1550 nm. A big advantage of implementing a resonant enhanced waveguide amplifier system using this rare earth doped material system lies it the much smaller wavelength spacing between the pump and signal, and therefore, the aforementioned issues associated with the 980 nm/1550 nm system are less severe.

In this paper, we demonstrate ring resonators fabricated in silicon rich nitride waveguides which are single mode at 1550 nm, but which support multiple modes at 1310 nm. Experimental characterization shows that these resonators operate well at both wavelengths, with reasonably high quality factors exceeding 10,000. The existence of resonances from higher order modes at 1310 nm is also ruled out. Characterization of Dy-Er co-doped NaYF_4_ particles show that photoluminescence is achieved around 1500–1525 nm when pumped at 1310 nm. These resonators are therefore ideal for use as low power, resonant assisted waveguide amplifiers pumped at 1310 nm.

**Figure 1:**
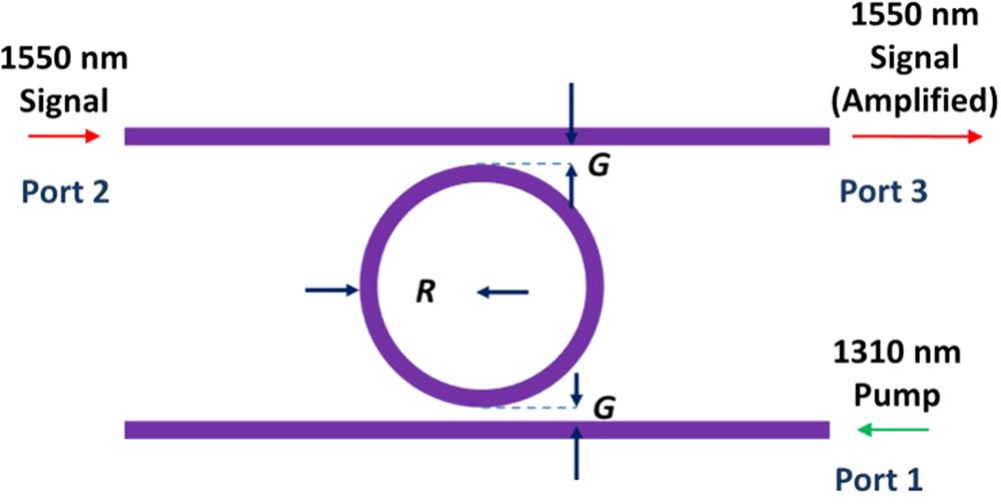
Configuration for resonator assisted amplification of light at 1550 nm using a pump at 1310 nm.

Figure 1 shows the device schematic for a system operating with a 1310 nm pump. A pump at 1310 nm is launched from port 1 and the signal at 1550 nm is launched at port 2. This allows both pump and signal to undergo an increased interaction time and therefore reducing the pump power required for amplification. The important condition is that both pump and signal wavelengths must be resonant within the resonator. In addition, to ensure that the pump efficiency is not compromised by higher order modes, minimal amounts of power should be coupled to higher order modes.

In order to realize the resonators for the aforementioned purpose of minimizing the amount of power needed to amplify light, we utilize silicon rich nitride on silicon dioxide as the waveguide platform. The silicon nitride film is deposited using low temperature chemical vapor deposition at a low temperature 250 °C which is compatible with back-end CMOS processing. The silicon rich nitride was characterized to have a linear refractive index of 3.1 which allows for more compact devices^29^. The bandgap of the silicon rich nitride is 2.05 eV, such that the two-photon absorption limit edge is below 1.3 µm which allows for minimal nonlinear losses at the operation wavelength. Because it is easily deposited using low temperature inductively coupled plasma chemical vapor deposition as an amorphous film, doping with rare earth materials within the silicon rich nitride matrix may be performed during the deposition. Alternatively, a polymer – based cladding containing the rare earth doped materials may be spincoated such that the evanescent tails of the propagating optical field may overlap with the gain material.

## Results

**Figure 2:**
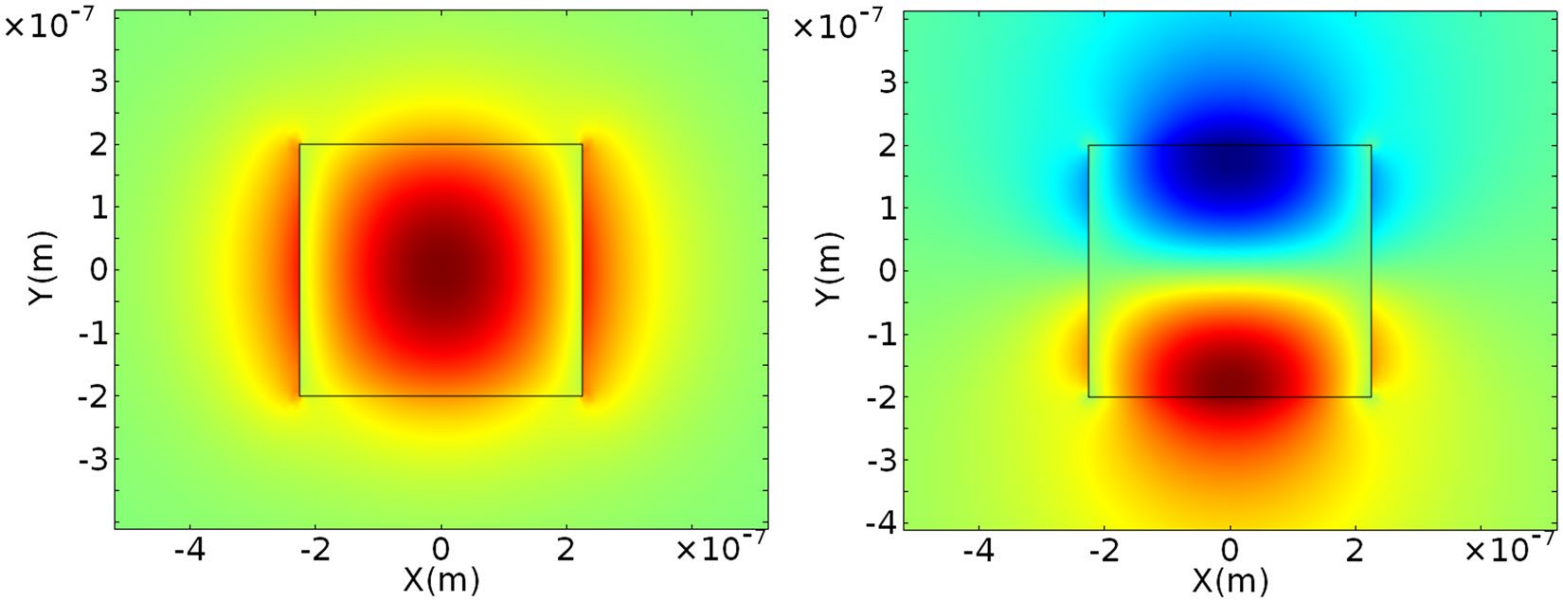
Calculated quasi – TE fundamental mode for the 400 × 450 nm waveguide at 1550 nm, and the first order quasi-TE mode for the waveguide at 1310 nm.

A channel waveguide configuration with *W* = 450 nm and *H* = 400 nm, with SiO_2_ cladding is studied first in this paper. At 1310 nm, 2 quasi - TE and TM modes exist whereas at 1550 nm, 1 quasi - TE and TM mode exists. The mode profiles of fundamental quasi-TE mode at 1550 nm and the first order quasi-TE mode of 1310 nm calculated using finite element modeling software are shown in Fig. 2. The fundamental quasi-TE mode profile of 1310 nm is similar to that of 1550 nm. For the TE light at 1310 nm, minimal light should be coupled from fundamental quasi-TE mode into first quasi-TE mode.

**Figure 3:**
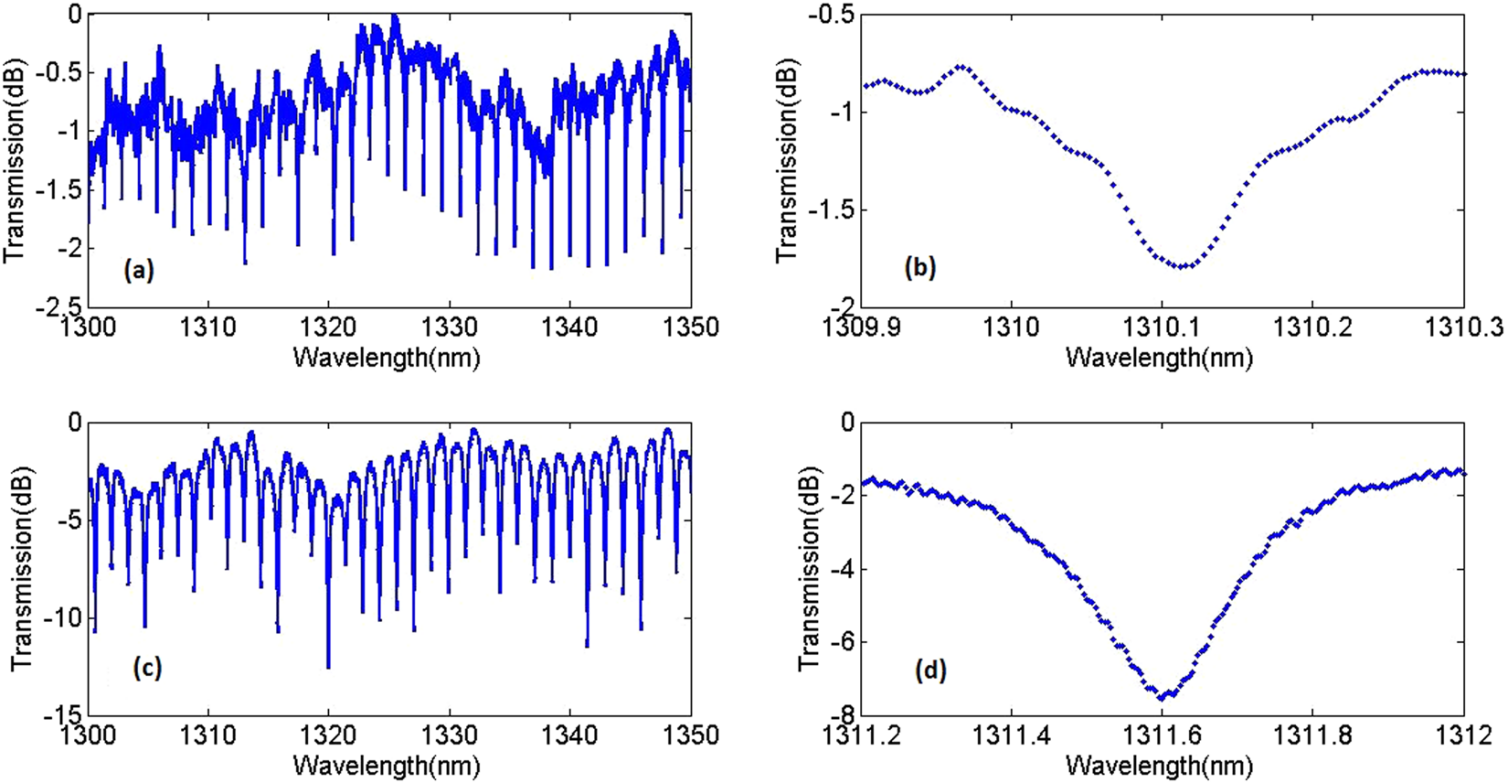
(**a**) Measured transmission spectrum at 1310 nm for a ring resonator with *R* = 50 µm and *G* = 200 nm for the quasi – TE mode of 400 nm × 450 nm waveguide. (**b**) Spectrum of a single resonance. (**c**) Measured transmission spectrum at 1310 nm for a ring resonator with *R* = 50 µm and *G* = 200 nm for the quasi – TM mode of 400 nm × 450 nm waveguide. (**d**) Spectrum of a single resonance.

The device fabrications and characterizations are described in the methods section. Figure 3 shows the measured transmission spectrum at 1310 nm for a ring resonator with *R* = 50 µm and *G* = 200 nm using both TE and TM light. The quasi-TE mode resonance has a higher quality factor than the quasi-TM mode, while the extinction ratio (ER) of quasi-TE mode is lower than that of the quasi-TM mode. The higher effective index of quasi-TE mode will result in lower propagation losses, and thus a higher quality factor. Because of the higher confinement of the quasi-TE mode compared to the quasi-TM mode, the gap width of 200 nm results in an undercoupled resonator and thus low extinction ratio, particularly if the gap is too large or the coupling region is insufficiently long.

**Figure 4:**
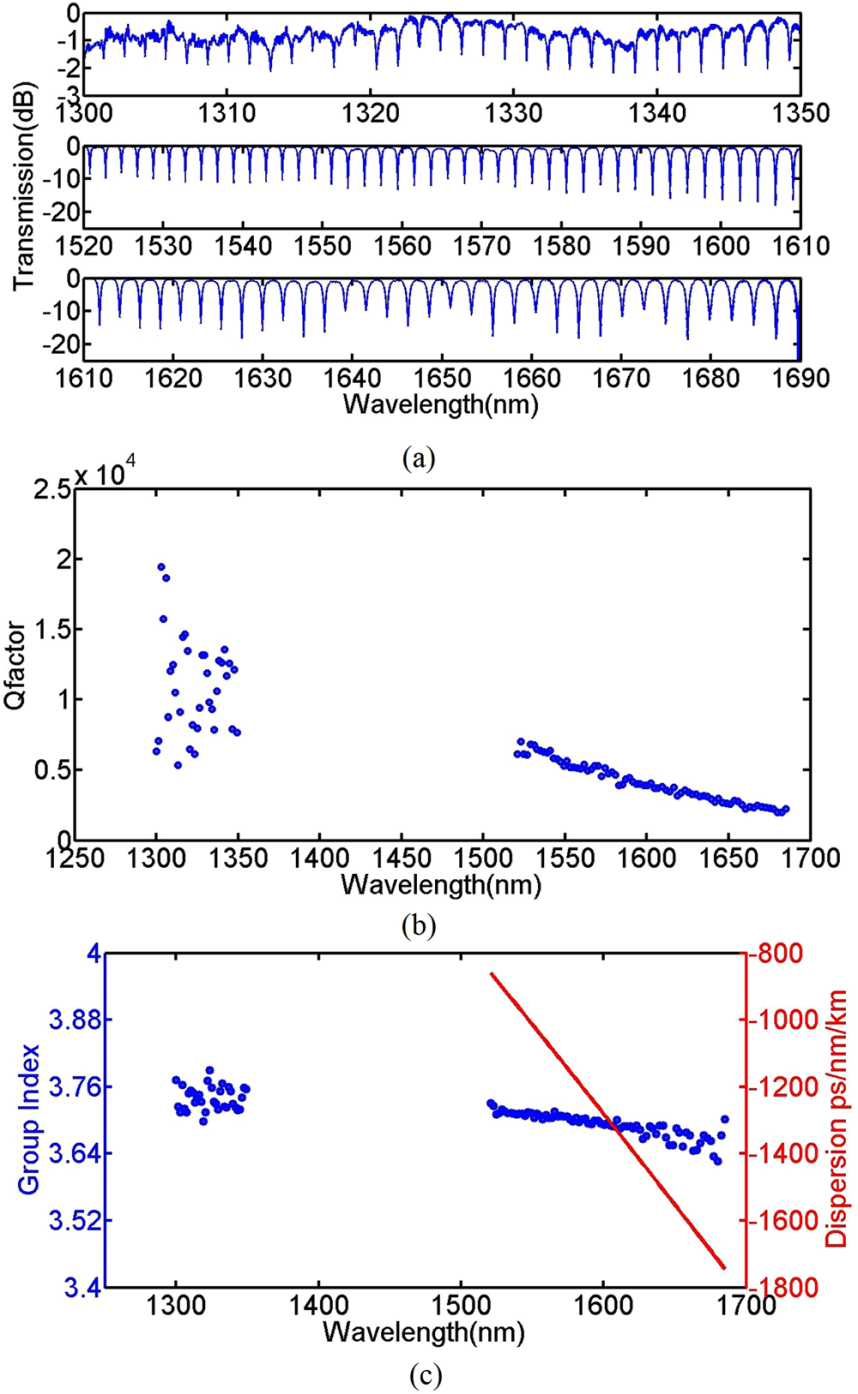
(**a**) Measured transmission spectrum, (**b**) quality factor and (**c**) waveguide group index (blue circles) and dispersion (red solid line) of the quasi – TE mode of a silicon rich nitride ring resonator, with *R* = 50 µm, *W* = 450 nm, *H* = 400 nm, *G* = 200 nm.

Figure 4a shows the transmission spectrum for the ring resonator with *R* = 50um and *G* = 200 nm for the quasi-TE mode between 1300–1350 nm and 1500–1690 nm.The quality factor of the resonators are extracted from the measured ring resonator spectra as shown in Fig. 4b using lorentzian fit. The calculated quality factors are from devices fabricated in the same fabrication run. The quality factor is observed to decrease with an increasing wavelength, which is the result of a lower effective index at higher wavelength. At 1300 nm, the quality factor is much higher. Resonances from higher order modes are also not observed in the measured spectrum, implying that minimal from the input fiber is coupled into the higher order mode at 1310 nm. Information about the group index and dispersion of the waveguide used to fabricate the ring resonators may also be derived from the ring resonator spectra, and the result is shown in Fig. 4c. From the transmission measured within the short wavelength range, it is observed that the group index is decreasing between 1500 nm – 1690 nm for the quasi – TE mode, implying normal dispersion. The extracted group velocity dispersion for 1500 nm – 1690 nm is also shown as the red curve in Fig. 4c.

To further investigate the performance of the ring resonators, ring resonators with different *W* and *G* are fabricated and characterized at both 1550 nm and 1310 nm. The quality factors for different ring resonators at 1550 nm and 1310 nm are listed in Tables 1 and 2 respectively. Results in Tables 1 and 2 represent the mean of quality factor of 5 resonance peaks near 1550 nm and 1310 nm respectively and are rounding to the nearest hundred. For some ring resonators, the extinction ratio (ER) is too low because of under-coupling, so the quality factors of those ring resonators are not analyzed. With increasing waveguide widths, the quality factor for ring resonator is observed to increase due to the reduced propagation loss and reduced coupling coefficient between the bus waveguide and ring. Also, while the gap *G* is increased, the quality factor increases because of the decreasing coupling coefficient of the coupler, implying a tradeoff between the quality factor and the extinction ratio in this regime.

**Table 1:** Quality factor for ring resonators using the quasi – TE mode with different W and G at 1550 nm.

*W*(nm)	450	500	550	600
*G*(nm)				
200	5,500	12,000	14,600	15,000
250	9,100	15,400	17,100	18,900
300	10,900	18,200	19,000	Low ER
350	16,000	Low ER	Low ER	Low ER

**Table 2:** Quality factor for ring resonators using the quasi – TE mode with different W and G at 1310 nm.

*W*(nm)	450	500	550	600
*G*(nm)				
200	9,600	17,000	Low ER	Low ER
250	12,900	Low ER	Low ER	Low ER
300	Low ER	Low ER	Low ER	Low ER
350	Low ER	Low ER	Low ER	Low ER

**Figure 5:**
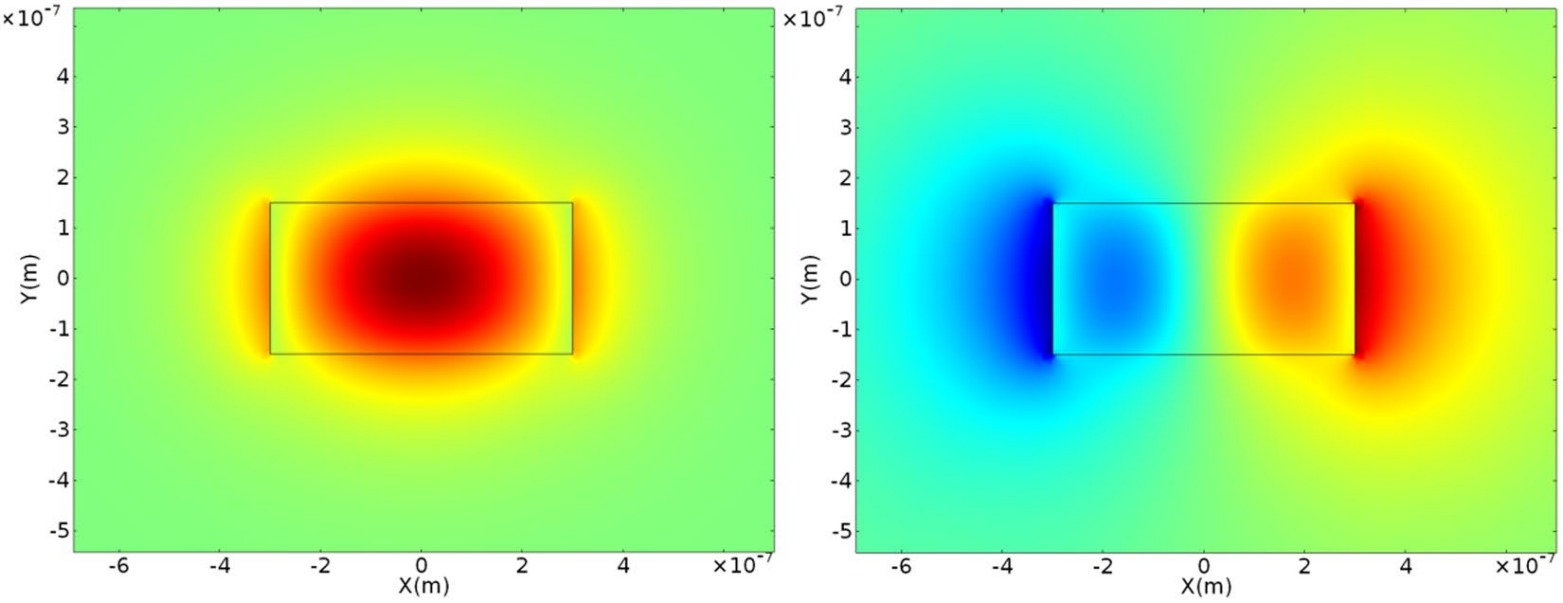
Calculated quasi – TE fundamental mode for the 300 nm × 600 nm waveguide at 1550 nm, and the first order quasi-TE mode at 1310 nm.

Another channel waveguide configuration with *W* = 550 nm and *H* = 300 nm, and SiO_2_ cladding is also studied. At 1310 nm, 2 quasi - TE and TM modes exist whereas the waveguide has 1 quasi - TE and TM mode at 1550 nm. This waveguide has a lower effective index for both the quasi-TE and quasi-TM mode compared to the 400 nm × 450 nm waveguide. The fundamental quasi-TE mode at 1550 nm and first quasi-TE mode at 1310 nm is plotted in Fig. 5. Compared with the 400 nm × 450 nm waveguide, this channel waveguide has a larger effective index difference between the fundamental and quasi-TE mode at 1310 nm. So the coupling from the fundamental mode into the higher mode will be reduced. And the quality factors of the ring resonators are expected to be higher at 1310 nm.

It is observed from Tables 1 and 2, that ring resonators with *H* = 400 nm, W = 500 nm and G = 250 nm possess quality factors of 17,000 and 12,000 at a wavelength of 1310 nm and 1550 nm respectively. This resonator design is therefore most ideal for hosting Dy-Er based amplifier systems utilizing a pump at 1310 nm for amplification at 1550 nm. To increase the quality factors, one possible approach is to reduce sidewall roughness induced through the fabrication process. The sidewall roughness can be minimized by optimizing the etch recipe or a short post-etching wet oxidation followed by an HF dip after etching^17^. Optimized post annealing step^30, 31^ has also been used for silicon nitride deposited by low pressure chemical vapor deposition to reduce its absorption. The same method, an optimized annealing step, can be deployed for the low temperature deposition to reduce the absorption of the silicon rich nitride.

**Figure 6:**
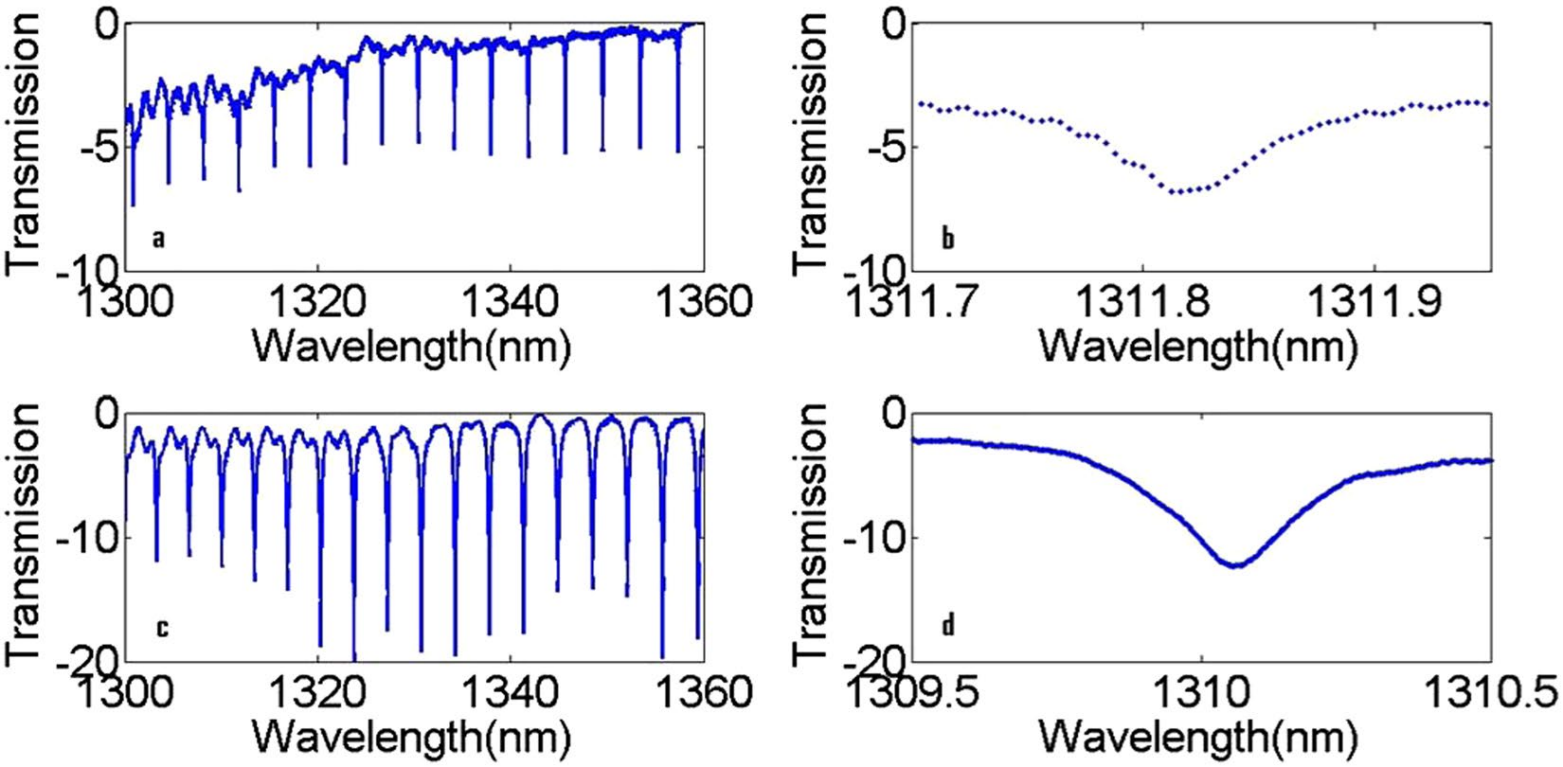
(**a**) Measured transmission spectrum at 1310 nm for a ring resonator with *R* = 20 µm and *G* = 150 nm for the quasi – TE mode of 300 nm × 600 nm waveguide. (**b**) Spectrum of a single resonance. (**c**) Measured transmission spectrum at 1310 nm for a ring resonator with *R* = 20 µm and *G* = 150 nm for the quasi – TM mode. (**d**) Spectrum of a single resonance.

We further characterize ring resonators fabricated on a 300 nm thick silicon rich nitride film with *W* = 600 nm, *G* = 150 nm, and *R* = 20μm. This ring resonator is characterized at the wavelength of 1310 nm using both the TE and TM light as described before. The transmission spectra are shown in Fig. 6. Similar to the first ring resonator, the TE mode has a higher quality factor but lower extinction ratio. The quality factor of the TE mode is around 14,000 which is higher than for the ring resonator with *W* = 450 nm, *H* = 400 nm and *G* = 200 nm. In addition, it also possesses a higher extinction ratio. The increased extinction ratio is due to the increased coupling coefficient resulting from the lower effective index of the waveguide and smaller gap *G*. So at the wavelength 1310 nm, this ring resonator has both higher extinction ratio and quality factor.

**Figure 7:**
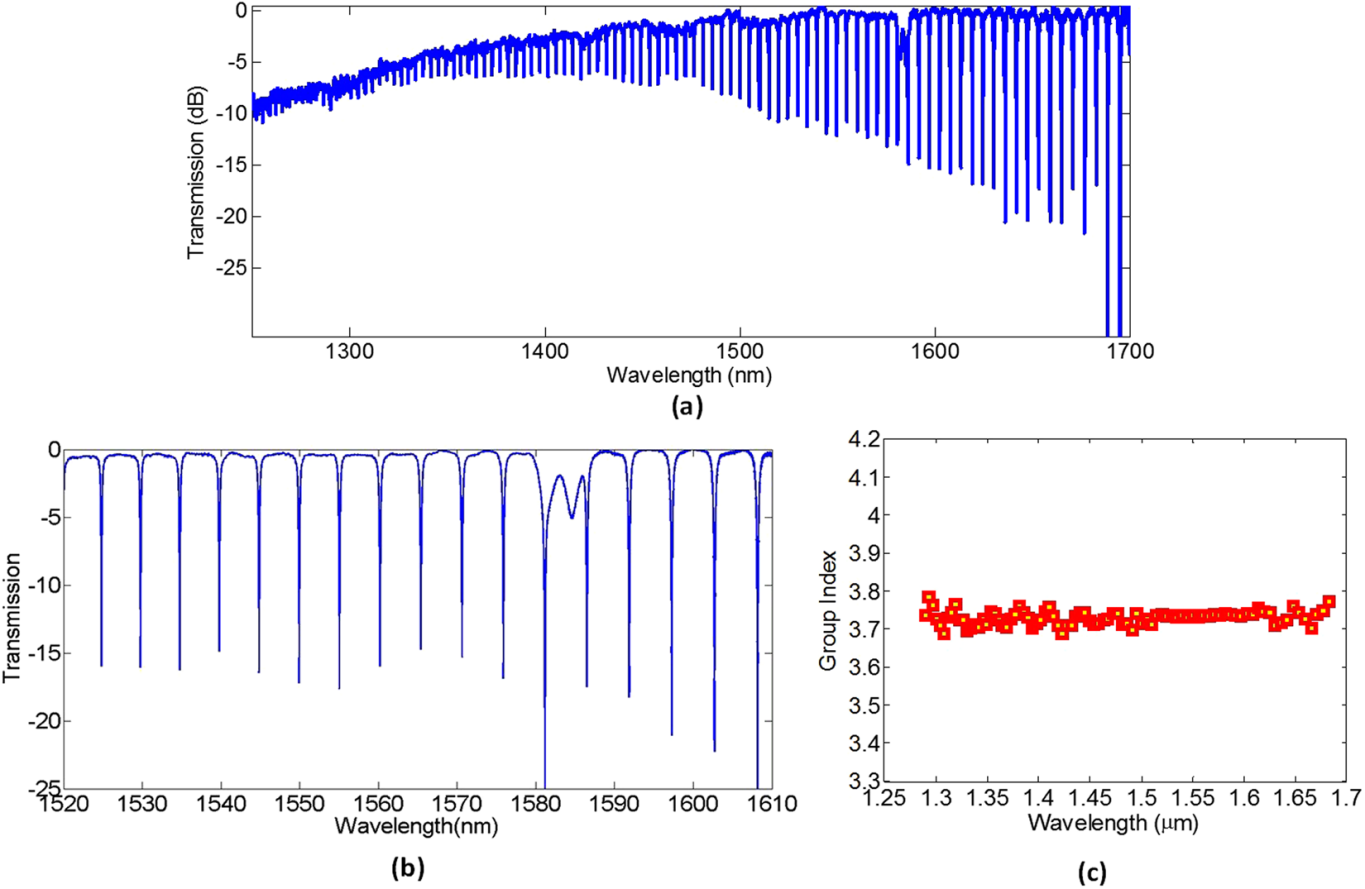
Transmission spectrum at 1550 nm for a ring resonator with *R* = 20 µm and *G* = 150 nm for the quasi – TE mode of 300 nm × 600 nm waveguide measured using (**a**) a supercontinuum source and (**b**) an amplified spontaneous emission source. (**c**) The measured group index as a function of wavelength extracted from (**a**).

The ring resonator is also characterized at 1550 nm using TE light as described before. The transmission spectrum is shown in Fig. 7. Figure 7a shows the transmission spectrum of the resonator measured using a supercontinuum source spanning from 1300 nm – 1700nm. The spectrum from 1520 nm–1610 nm measured using an ASE source is further shown in Fig. 7b. The FSR at 1550 nm is around 16.7 nm which is much higher than that of the previous ring resonator due to the smaller ring radius. The group index extracted from the spectrum is around 3.75 (see Fig. 7c). The quality factor around 1550 nm extracted from the spectrum is 5,100 which is similar to that of the first ring resonator. And the extinction ratio is higher. The increased extinction ratio is due to the decreased gap and waveguide effective index.

**Figure 8:**
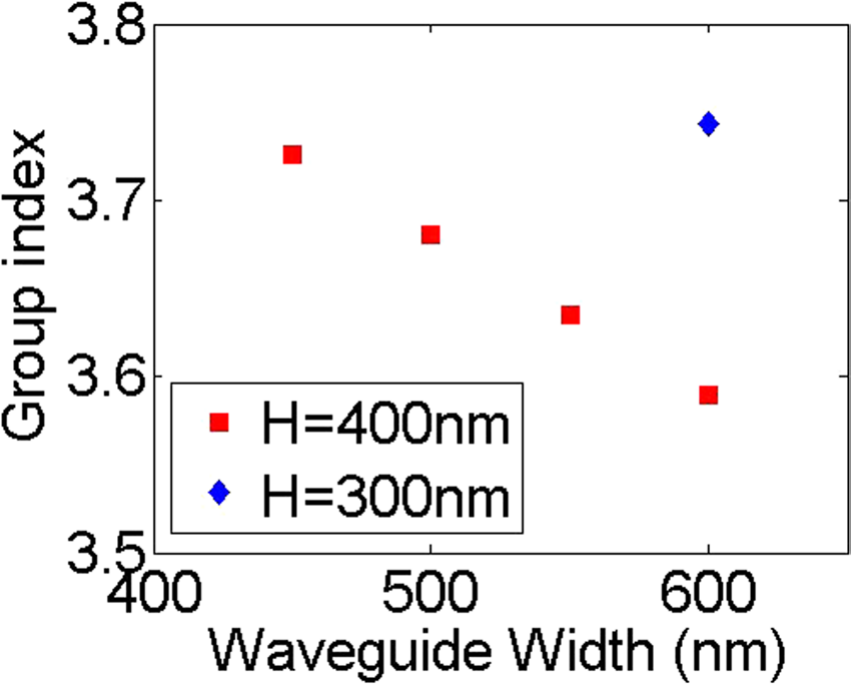
Group index for the channel waveguides with different width (*W*) and height (*H*).

Figure 8 summarizes the group index of the waveguides with different width (*W*) and height (*H*) at 1550 nm, derived from the measured ring resonator spectrum. The group index of the waveguides will decrease with increasing the waveguide width or height. It is observed that waveguide geometries where light is less confined within the waveguide core results in higher group indices.

**Figure 9:**
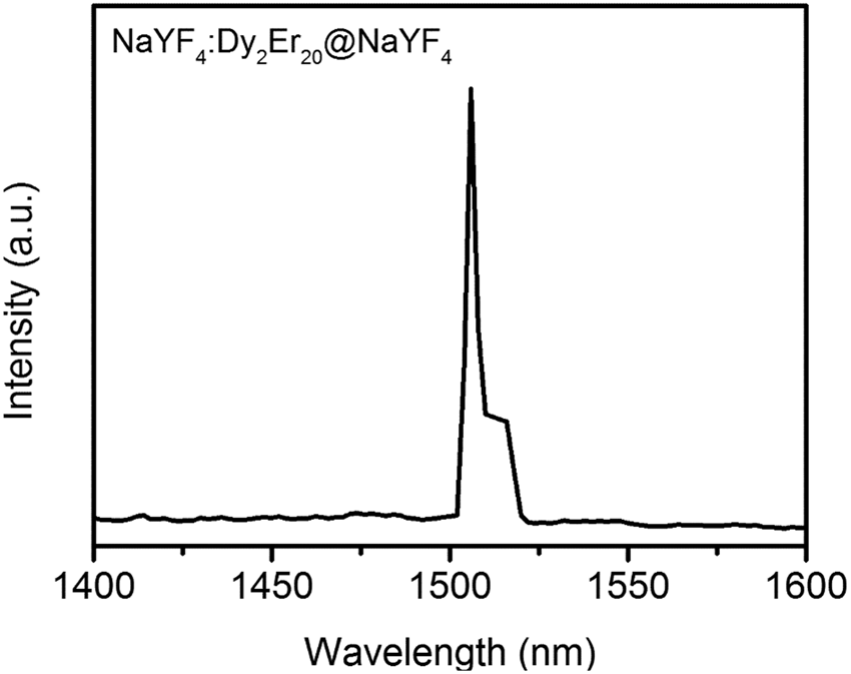
Photoluminescence at 1500–1525 nm when Dy-Er doped NaYF_4_ core-shell nanoparticles are pumped at 1310 nm.

**Figure 10:**
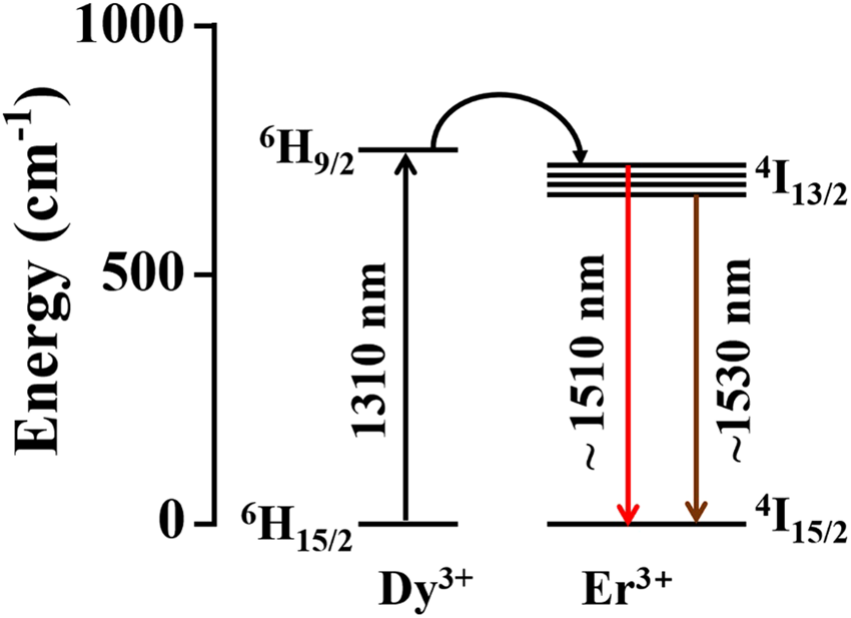
Energy transfer of Dy-Er ions while pumped at 1310 nm.

Dy-Er doped NaYF_4_ core-shell nanoparticles are synthesized using the thermal decomposition method as described elsewhere^32^. The core comprises of an actively emitting NaYF_4_ host co-doped with 20 mol% of Er and 2 mol% of Dy, whilst an undoped shell of NaYF_4_ is coated around the core particle to prevent undesired surface quenching effects that results in the reduction of emissions. The steady state emission spectra were measured at room temperature upon excitation with a 1310 nm continuous wave laser (SDL-1310-LM-700MFL, Shanghai Dream Lasers Technology Co., Ltd.) using a FLS980 Fluorescence Spectrometer (Edinburgh Instruments Ltd., UK) spectrometer equipped with near infrared detector (Hamamatsu H1033A-75). For infrared emission measurements, a step size of 2 nm with dwell time of 0.2 second is used for the laser at 37 mW with a spot size of 19.63 mm^2^. The 300 mm focal length monochromator was used. The grating for infrared emission is 1200 nm (700–1800 nm). The powder samples were packed in demountable spectrosil far UV quartz Type 20 cells (Starna Cells, Inc., Atascadero, CA) with 0.5 mm path lengths for emission collection. It is observed from Fig. 9 that photoluminescence at 1500 nm-1525 nm is present. As shown in Fig. 10 ^33^, the emission comes from transitions of electrons from the top energy level in the fine energy structures of ^4^I_13/2_ state to the ^4^I_15/2_ state. By changing the host materials, the doped materials can also emit at ~1530 nm which is from the lower energy level of ^4^I_13/2_ state.

Integrating these particles within the SRN matrix either during the film deposition step, or post fabrication as a polymer based upper cladding could lead to O-band pumped amplifiers for the telecommunications-band, leveraging resonators with reasonably high quality factors at both pump and signal wavelengths.

## Discussion

In the Dy-Er co-doped systems, the pump is located at ~1310 nm and the signal at ~1550 nm. Because of the broad gain spectrum, wavelengths around 1550 nm can be amplified with varying gain coefficients. In order to access the viability of our ring resonators for amplification purposes, it is useful to analyze the resonator quality factors. The significance of the quality factor at 1310 nm lies in the build-up of the pump’s optical energy within the resonator. Since the gain of the system relies strongly on the amount of pump power, higher quality factors generate an enhancement of optical energy useful for realizing higher gain per unit length. The second important factor is the quality factor at 1550 nm. In this case, a high quality factor is desirable in order to generate a longer effective path length. Since the gain is proportional to the optical path length traveled, a higher quality factor at 1550 nm would lead to a higher overall gain.

Within the resonators characterized, the resonator with *H* = 400 nm, W = 500 nm and *G* = 250 nm possesses quality factors of 17,000 and 12,000 at a wavelength of 1310 nm and 1550 nm respectively. For the first ring resonator fabricated on 400 nm silicon rich nitride, the finesse of the resonator at 1310 nm is ~20, which implies a power enhancement of ~6.4. The finesse at 1.55um is ~15.5, which implies a power enhancement of ~5. However, the more important metric at 1550 nm is the effective optical path length, which may be calculated using the quality factor of 12,000 to be 4.9 mm. For the second ring resonator fabricated on 300 nm silicon rich nitride, due to the much larger FSR which is 16.7 nm, the finesse of the resonator at 1.55um is ~17.5, which implies a power enhancement of ~5.6. The effective optical path length using the quality factor of 5100 works out to be 2.2 mm. At 1310 nm, the finesse of the resonator is ~40, which implies a power enhancement of ~12.7.Consequently, these resonators could allow low power, Dy-Er based amplifiers pumped at 1310 nm for amplification of light at 1550 nm to be realized. Specifically, the resonator with *H* = 400 nm, W = 500 nm and G = 250 nm possesses quality factors of 17,000 and 12,000 at a wavelength of 1310 nm and 1550 nm respectively, and could enable the pump field at 1310 nm to be significantly enhanced, while also enabling an augmented optical path length for the traveling 1550 nm field to be amplified. The same resonator however, exhibits poor extinction at 1 µm, and therefore is not suitable for used as a dual – wavelength resonator necessary in Er – doped amplifiers with a 980 nm pump.

## Conclusions

In conclusion, we proposed a rare-earth doped amplifier based on silicon rich nitride ring resonators with Dy-Er NaYF_4_ particles. The ring resonators are fabricated and characterized over a wide spectral range covering both 1310 nm and 1550 nm. Ring resonators exhibiting quality factors exceeding 10,000 simultaneously at 1310 nm and 1550 nm are demonstrated, and provide a promising platform for low power Dy-Er based amplifiers pumped at the O-band for signal amplification at the telecommunications-band.

## Methods

### Device Fabrications

To fabricate the ring resonators, silicon rich nitride films are first deposited using inductively coupled chemical vapor deposition. The deposition temperature is 250 °C which makes it compatible with back-end CMOS processes. SiH_4_ and N_2_ are used as precursor gases with a ratio of 2:1. Electron – beam lithography and reactive ion etching is then used to pattern and etch the waveguides followed by a deposition of SiO_2_ using plasma enhanced chemical vapor deposition (PECVD).

### Measurements

Ring resonators are characterized at both 1310 nm and 1550 nm. Two broadband lasers at 1310 nm and 1550 nm are utilized as light sources. The output light from laser transmit through a polarized-maintained fiber maintaining linearly polarized. Then light polarization direction is adjusted to be horizontal to maximize the quasi-TE mode coupling efficiency or vertical to maximize the quasi-TM mode coupling efficiency. Tapered lensed fibers and inverse tapers on the waveguide side are used to minimize fiber – waveguide coupling losses. The measured coupling loss per taper is around 7 dB. The transmission spectrum is obtained using optical spectrum analyzer.
